# Modulation of Gut Microbiota by Magnesium Isoglycyrrhizinate Mediates Enhancement of Intestinal Barrier Function and Amelioration of Methotrexate-Induced Liver Injury

**DOI:** 10.3389/fimmu.2022.874878

**Published:** 2022-05-12

**Authors:** Yawen Xia, Hang Shi, Cheng Qian, Hongkuan Han, Keqin Lu, Ruizhi Tao, Renjun Gu, Yang Zhao, Zhonghong Wei, Yin Lu

**Affiliations:** ^1^Jiangsu Key Laboratory for Pharmacology and Safety Evaluation of Chinese Materia Medica, School of Pharmacy, Nanjing University of Chinese Medicine, Nanjing, China; ^2^Jiangsu Joint International Research Laboratory of Chinese Medicine and Regenerative Medicine, Nanjing University of Chinese Medicine, Nanjing, China; ^3^Jiangsu Collaborative Innovation Center of Traditional Chinese Medicine (TCM) Prevention and Treatment of Tumor, Nanjing University of Chinese Medicine, Nanjing, China; ^4^Jiangsu Provincial Second Chinese Medicine Hospital, The Second Affiliated Hospital of Nanjing University of Chinese Medicine, Nanjing, China; ^5^Department of Biochemistry and Molecular Biology, School of Medicine & Holistic Integrative Medicine, Nanjing University of Chinese Medicine, Nanjing, China

**Keywords:** methotrexate, magnesium isoglycyrrhizinate, gut-liver axis, bacterial translocation, gut microbiota

## Abstract

**Background:**

The gut–liver axis plays a crucial role in various liver diseases. Therefore, targeting this crosstalk may provide a new treatment strategy for liver diseases. However, the exact mechanism underlying this crosstalk and its impact on drug-induced liver injury (DILI) requires clarification.

**Aim:**

This study aimed to investigate the potential mechanism and therapeutic effect of MgIG on MTX-induced liver injury, which is associated with the gut–liver axis and gut microbiota.

**Methods:**

An MTX-induced liver injury model was generated after 20-mg/kg/3d MTX application for 30 days. Meanwhile, the treatment group was treated with 40-mg/kg MgIG daily. Histological examination, aminotransferase, and aspartate aminotransferase enzyme levels were estimated to evaluate liver function. Immune cells infiltration and inflammatory cytokines were detected to indicate inflammation levels. Colon histological score, intestinal barrier leakage, and expression of tight junctions were employed to assess the intestinal injury. Bacterial translocation was observed using fluorescent *in situ* hybridisation, colony-forming unit counting, and lipopolysaccharide detection. Alterations in gut microbial composition were analysed using 16s rDNA sequencing and relative quantitative polymerase chain reaction. Short-chain-fatty-acids and lactic acid concentrations were then utilized to validate changes in metabolites of specific bacteria. *Lactobacillus* sp. supplement and fecal microbiota transplantation were used to evaluate gut microbiota contribution.

**Results:**

MTX-induced intestinal and liver injuries were significantly alleviated using MgIG treatment. Bacterial translocation resulting from the intestinal barrier disruption was considered a crucial cause of MTX-induced liver injury and the therapeutic target of MgIG. Moreover, MgIG was speculated to have changed the gut microbial composition by up-regulating probiotic *Lactobacillus* and down-regulating *Muribaculaceae*, thereby remodelling the intestinal barrier and inhibiting bacterial translocation.

**Conclusion:**

The MTX-induced intestinal barrier was protected owing to MgIG administration, which reshaped the gut microbial composition and inhibited bacterial translocation into the liver, thus attenuating MTX-related DILI.

**Graphical Abstract:**
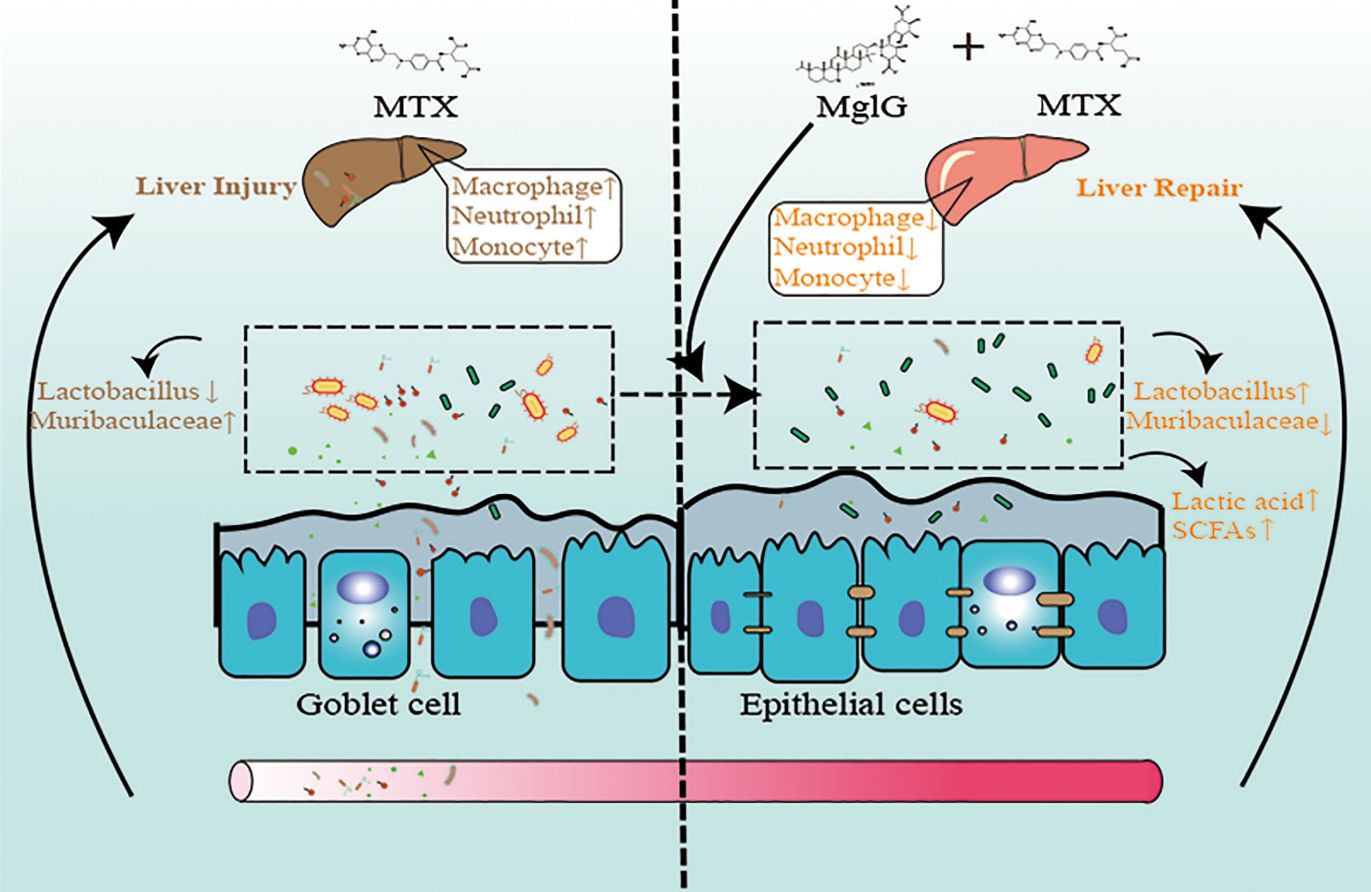
Schematic illustration of the therapeutic effects of magnesium isoglycyrrhizinate (MgIG) on methotrexate (MTX)-induced intestinal and liver injuries: MTX induces intestinal barrier damage by altering the microbiota composition, which causes bacterial translocation and liver inflammation. However, MgIG treatment elevated Lactobacillus levels and restored the intestinal barrier, which acts as a protective barrier in MTX-induced liver injuries.

## Introduction

Methotrexate (MTX), a folic acid antagonist, has been clinically used to treat inflammatory arthritis and various cancers, especially acute lymphoblastic leukaemia (ALL) and breast cancer (1, 2). Hepatotoxicity-induced fatty infiltration and fibrosis, which can lead to cirrhosis, is a severe effect of prolonged MTX administration (3–5), thus limiting its clinical application. Furthermore, MTX administration has been associated with the risk of increased alanine aminotransferase/aspartate aminotransferase (ALT/AST) enzyme levels in patients with rheumatoid arthritis (RA) or psoriatic arthritis (PsA) (6, 7). However, the underlying mechanisms of MTX hepatotoxicity remain unclear.

Gastrointestinal reactions, which are other common MTX complications, are characterised by the impaired structure and function of the mechanical barrier of the intestinal mucosa (8, 9). The impaired function of the intestinal barrier function contributes to bacterial translocation across the barrier (10, 11), including bacterial toxic metabolites, and migration into the blood circulation, lymphatic system, and liver *via* the entero-hepatic circulation, which can induce liver injury (12). Various studies have reported the importance of this crosstalk in different liver diseases (13–15), indicating that gastrointestinal and liver injuries could be caused by MTX. Therefore, this hypothesis was validated using different time points designed to observe whether intestinal injury preceded hepatic lesion development.

Magnesium isoglycyrrhizinate (MgIG), which is synthesised from 18-β glycyrrhizic acid, has been clinically administrated for various liver disease treatments, including drug-induced liver injury (DILI), non-alcoholic fatty liver disease (NAFLD), autoimmune liver disease, and cirrhosis (16–19). Multiple systematic reviews and trials have demonstrated that hepatotoxicity grade I incidence and alanine aminotransferase level were found to be significantly reduced in patients who were administered MgIG compared to those who were administered standard care glutathione or tiopronin (17, 20, 21). Moreover, in addition to alleviating the liver injury, MgIG has been reported to significantly reduce intestinal permeability and inflammation caused by MTX in rats (22, 23). Therefore, the association between these two protective effects, which is based on the gut–liver axis theory, and the potential mechanisms and therapeutic effects of MgIG in DILI were speculated. This study reveals that MgIG, a prebiotic analogue, alters microbial ecology, which contributes to intestinal barrier integrity and alleviates MTX-caused liver injuries (22).

## Methods and Materials

### Animal Treatment

The 8-week-old male mice required for the experiments were purchased at Life River Laboratory Animal Technology Ltd (Beijing, China) and housed in a Specific Pathogen Free Facility (SPF) which was maintained at 23 ± 1°C and 50% humidity with a dark-light cycle of 12 h:12 h (lights on at 07:30). All experimental protocols were approved by the Animal Care and Use Committee of Nanjing University of Chinese Medicine (Nanjing, China) and were conducted following the Guidelines for the Care and Use of Laboratory Animals (201904A007, 202102A011). MTX-induced DILI model was established using 20mg/kg/3days administration of MTX by intraperitoneal injection. To determine the protective effect of MgIG, C57BL/6 mice were treated with intraperitoneal injection of MgIG 40mg/kg once a day for total of 4 weeks.

### Gut Flora Identification

Fecal samples of mice were collected in steriled tubes and stored at −80°C before further processing. After using QIAamp Fast DNA Stool Mini Kit (Qiagen, USA) to extract genomic DNA from samples, 1% agarose gel was used to detect DNA concentrations and monitor purity. 16S rRNA genes were amplified using specific primer with the barcode was used to amplify 16S rRNA genes. All PCR reactions were carried out with TransStart FastPfu DNA Polymerase (TransGen, Beijing, China). The universal amplification primers of bacterial 16S rRNA gene are as follows: forward primer was 5’-CCTACGGGNGGCWGCAG-3’ and reverse primer was 5’-GACTACHVGGGTATCTAATCC-3’. GeneJET Gel Extraction Kit (Thermo Fisher Scientific, USA) was used to purify the mixture of PCR products. Following manufacturer’s recommendations, sequencing libraries were generated using NEB Next Ultra DNA Library Prep Kit for Illumina (NEB, USA) and index codes were added. The library was sequenced on an Illumina MiSeq platform. Sequence alignment, operational taxonomic units (OTUs) picking against the SILVA reference collection, clustering, phylogenetic and taxonomic profiling, and the analysis of beta diversity were performed with the Quantitative Insights into Microbial Ecology (QIIME) software package. Differential genera bacteria were identified using Kruskal-wallis H test and LEfSe analysis.

### H&E Staining and Histopathologic Analysis

Liver and colon tissues were randomly selected (n=6 per group). Liver sections were cut into pieces and fixed using 4% paraformaldehyde. Colon sections were slitted and fixed in Carnoy’s fixative overnight. The liver and colon biopsy samples of mice were placed in their respective processing cassettes, dehydrated *via* a serial alcohol gradient, and embedded in paraffin wax blocks. Before immunostaining using H&E, tissue sections were dewaxed in xylene, rehydrated using decreasing ethanol concentrations, and washed using 1x phosphate-buffered saline (PBS). After staining, the sections were dehydrated using increasing concentrations of ethanol and xylene. The histological score of the liver was evaluated using the Ishak system (**Supplementary Table S1**). Histological scores were further used to assess the degree of inflammatory infiltrate and epithelial damage in various colon tissues, including the submucosa, mucosa, muscularis, and serosa. A total scoring range of 0 to 12 per mouse was added by four scores per colon. Blinded pathobiological examination was conducted.

### RNA Isolation and q-PCR

Total RNA was isolated from either cells or organoids using the TRIzol reagent according to the manufacturer’s instructions. Reverse transcription of RNA into cDNA was performed using a Hiscript^®^ ll Q RT SuperMix (Vazyme, Shanghai, China) for qPCR (+gDNA wiper). The cDNA was amplified with specific primers to detect target mRNA expression using a ChamQTM SYBR^®^ qPCR Master Mix (Low ROX Premixed). Glyceraldehyde 3-phosphate dehydrogenase was used as a reference gene for relative quantification. The primer sequences used were as follows.

GAPDH-forward, AGG TCG GTG TGA ACG GAT TTG

GAPDH-reverse, TGT AGA CCA TGT AGT TGA GGT CA

TNF-α-forward, GTT CTG TCT ACT GAA CTT CGG G

TNF-α-reverse, GAG GCT TGT CAC TCG AAT TTT G

ZO-1-forward, GGG CCA TCT CAA CTC CTG TA

ZO-1-reverse, AGA AGG GCT GAC GGG TAA AT

IL-1β-forward, ACG GAC CCC AAA AGA TGA AG

IL-1β-reverse, TTC TCC ACA GCC ACA ATG AG

IL-6-forward, CCA GTT GCC TTC TTG GGA CT

IL-6-reverse, GGT CTG TTG GGA GTG GTA TCC

Occludin-forward, ACT ATG CGG AAA GAG TTG ACA G

Occludin-reverse, GTC ATC CAC ACT CAA GGT CAG

Claudin-1-forward, GAA TTC TAT GAC CCC TTG ACC C

Claudin-1-reverse, TGG TGT TGG GTA AGA GGT TG

IL-10-forward, GCT CTT ACT GAC TGG CAT GAG

IL-10-reverse, CGC AGC TCT AGG AGC ATG T

E-cadherin-forward, AAG ACC AGC GGC GTA TTG GA

E-cadherin-reverse, CAG CAC CAG TGG AAC CGA CG

IL-4-forward, TGA ACG AGG TCA CAG GAG AAG

IL-4-reverse, TTG GAA GCC CTA CAG ACC AG

IFN-γ-forward, AAC AAA GGC TCT GGA GGG CTC

IFN-γ-reverse, TGA TAG GCG GTG AGG CTA CA

TLR4-forward, GTA TTG CCA AGT TTG AGG TCA AC

TLR4-reverse, GCT TCC TGA GGC TGG ATT C

MCP-1-forward, ACA GAC AGA GGC CAG CCC AG

MCP-1-reverse, TCT CCA GCC GAC TCA TTG GGA

### Fecal DNA Extraction and Quantification of *Lactobacillus* sp.

E.Z.N.A Stool DNA KIT (Omega Bio-tek, USA) was used to extract Total fecal DNA. qPCR on a 7500 Sequence Detector (Applied Biosystems, CA, USA) was used to calculate the number of Lactobacillus sp. Relative gene copy number of Lactobacillus in genomic DNA extracted from fecal samples were calculated by the qPCR on a 7500 Sequence Detector (Applied Biosystems, CA, USA) and compared with 16s rRNA gene.Samples were quantified in 20 μl reactions using ChamQ SYBR qPCR Master Mix (Low ROX Premixed) (Vazyme, Shanghai, China). Lac-F (5’-TGGAAACAGGTGCTAATACCG-3’) Lac-R (5’- CCATTGTGGAAGATTCCC-3’) 16S rRNA-F (5’-ACTCCTACGGGAGGCAGCAGT-3’) 16S rRNA-R(5’-GTATTACCGCGGCTGCTGGCAC-3’). All measurements were performed in duplicate.

### Lactic Acid Concentration Measurement of Faecal Samples

A total of 100-mg colon contents were homogenised with 0.9 mL of PBS followed and then centrifuged at 5000 g for 15 min. The supernatants were transferred and stored at -80°C until further experimentation. According to the manufacturer’s instructions, a Lactic acid Detection Kit (Jinyibai Biological Technology Co., Ltd., Nanjing, China) was used to assess the lactic acid concentration which was normalised using colon content weight.

### *Lactobacillus* sp. Culture

*Lactobacillus* sp. (BNCC189810, purchased from (BeNa Culture Collection, China) was cultured in Man, Rogosa, and Sharpe (MRS) broth (Solarbio, China) at 37°C under anaerobic conditions (80%N2, 10%CO2, 10%H2) according to ATCC in a micro-anaerobic incubation system (ELECTROTEK, AW500SG/TG).

### Western Blotting

Proteins were lysed from tissues or cells using radioimmunoprecipitation assay buffer, which contains phosphatase and protease inhibitors. Following this, protein samples were separated using sodium dodecyl sulfate–polyacrylamide gel electrophoresis and transferred onto polyvinylidene fluoride (PVDF) membranes. The PVDF membranes were incubated in tris-buffered saline with 0.1% Tween 20 (TBST), containing non-fat milk, for 2 h. The membranes were incubated with the primary antibody overnight. The primary antibodies of ZO-1 (Affinity Biosciences LTD.), E-cadherin (Cell Signaling Technology), and Claudin-1 (Affinity Biosciences LTD.) were used. After three rinses with TBST, the membranes were incubated with the secondary antibody. Cruz Marker™ MW (Santa Cruz) was used for indicating the molecular weights and protein signal was detected using the ChemiDocTM Touch Imaging System (BIO-RAD).

### Immunohistochemistry

Immunohistochemical analyses of the healthy/injured liver were performed on 5-μm-thick tissue sections that underwent antigen retrieval using citrate buffer solution. The sections were blocked using 5% bovine serum albumin solution. Following this, the primary antibodies, which included anti-CD45 (Cell Signaling Technology) and anti-TLR4 (Affinity Biosciences LTD.) were incubated with the tissue sections overnight at 4°C. The sections were then incubated with Horseradish Peroxidase (HRP) secondary antibodies (Proteintech, China) according to the corresponding sources. The sections were developed using a diaminobenzidine substrate kit and counterstained using haematoxylin. Tissue images were obtained using a microscope.

### Immunofluorescence

Immunofluorescence analysis was performed on tissue sections at 5 μm thickness and underwent antigen retrieval using citrate buffer solution. Primary antibodies included anti-E-cadherin (Affinity), anti-Claudin-1 (Affinity), and anti-mucin-2 (Affinity) antibodies were incubated with colon tissue sections, and anti-CD68 (Abcam), anti-iNOS (Abcam), anti-CD163 (Abcam) for liver tissues overnight at 4°C. Then the sections were incubated with secondary antibodies according to the corresponding sources as follows: Alexa Fluor 594 conjugated Donkey anti-Mouse and Alexa Fluor 488 conjugated Goat anti-Mouse. Images were obtained by a fluorescence microscope (Zeiss).

### Periodic Acid-Schiff Staining (PAS)

Before immunostaining using PAS, tissue sections were dewaxed in xylene and washed with 1x PBS. Then tissues were handled with Periodate alcohol solution, Schiff’s solution, and Harris hematoxylin. After staining, the tissues were dehydrated using increasing concentrations of ethanol and xylene. Tissue images were obtained using a microscope.

### Flow Cytometry on Liver Samples

Liver samples were flushed and rinsed in cold PBS and then transferred into collagenase D solution (1 mg.mL^-1^) (BioFroxx) at 37°C for 30 min. The reaction was stopped by adding cold ethylenediaminetetraacetic acid (EDTA) containing flow cytometry staining buffer (PBS, 10% FBS, 0.1% NaO3 and 5-mM EDTA). Since there are many Fc receptors on the surface of macrophages and monocytes, Fc receptors were blocked using TruStain FcX™ (anti-mouse CD16/32) Antibody (Biolegand) at 1.0 µg per 10 (6) cells in 100 µl volume for 5-10 min on ice prior to immunostaining. Then, liver tissues were stained with anti-CD45-FITC (Biolegand), anti-CD11b-APC (Biolegand), anti- Ly6C-PE (Biolegand), anti-Ly6G-PerCp/Cy5.5 (Biolegand) and anti-F4/80-PE/Cy7 (Biolegand) at 4°C in the dark for 40 min. Beckman Cytoflex was used for cell estimation. About 30000 cells were recorded and 10000 cells were shown in the figures. Gating method is attached in **Supplementary Figure S7**.

### Measurement of Cytokines in the Serum

The concentration of cytokines and chemokines in the serum was quantified using the MILLIPLEX MAP Mouse Cytokine/Chemokine Magnetic Bead Panel (MCYTOMAG-70K-10, Millipore), containing IL-1β, MCP-1(CCL2), MIP-2(CXCL2), IL-17, TNF-α, IFN-γ, IL-4, IL-10, IL-6 and GM-CSF, according to the manufacturer’s instructions.

### Fluorescent *In Situ* Hybridization (FISH)

FISH analysis of the healthy/injured liver was performed on 5-μm-thick tissue sections. Tissue sections were washed using PBS for 15 min and blocked using a blocking buffer for 2 h at 55°C. The probe dilution (probe: hybridisation buffer = 1:50-200) was denatured at 88°C for 3 min and balanced at 37°C for 5 min. Following this, the probe dilution was added to the sections and hybridised for 16–72 h at 37°C–42°C (in a dark place). Finally, the sections were washed using a washing buffer for 15 min after hybridisation.

### Assessment of Bacterial Translocation

Liver slices were collected and weighed under sterile conditions and then washed in sterile PBS for 10 s. Transfer to sterile PBS containing 5% fetal bovine serum and 0.5 mg/ml gentamicin and culture in gentamicin at 37°C for 30 min. The samples were then washed and dissolved in PBS. The dissolved tissues were inoculated on sterile sheep blood Columbia agar (Solarbio, China) plates and cultured under aerobic conditions at 37°C for 48 h. After 48 h of culture, colony-forming units (CFUs) were counted and CFUs per gram of tissue were measured.

### Measurement of FITC-Dextran Leakage

FITC-dextran leakage was conducted as reported (24). Mice were fasted overnight for 6 h and gavaged with FITC-dextran (Sigma-Aldrich) at a concentration of 50 mg/ml. In short, after 3 h, the mice were anesthetized with isoflurane and imaged *in vivo* with IVIS Lumina series III (Perkin Elmer). Serum was collected 4 h after administration of FITC-Dextran and measured using a fluorescence microplate reader (Perkin Elmer). FITC dextran was used to obtain the standard curve and evaluate the dextran concentration in serum.

### Fecal Microbiota Transplantation

Fecal transplantation was performed according to an established protocol (25). Briefly, stools from control mice or MgIG-treated or untreated MTX models were collected, snap-frozen, and stored at -80°C. Stools were collected of donor mice in each group, 100mg was taken and suspended in 1ml sterile saline. Shaken for 10 s, the solution was mixed evenly, centrifuged at 800°C for 3 min, the supernatant was collected and used for transplantation. On the day of transplantation, fresh transplantation materials were prepared within 10 min before oral gavage to prevent changes in bacterial composition. Before fecal microbiota transplantation, mice were fed with antibiotic water for 5 days which was composed of 1g/L ampicillin, 1g/L metronidazole, 0.5 g/L vancomycin, and 0.5 g/L neomycin. Transplantation was performed by oral gavage of 200 μl transplant material once per week.

### Statistical Analyses

All statistical analyses were carried out with the GraphPad Prism software version

(GraphPad, San Diego, CA). The data are presented as mean ± standard deviation (SD). The data were analysed using two-tailed Student’s t-test between two groups, one-way analysis of variance and Dunnett’s *post hoc* tests when groups were more than two. P < 0.05 was considered statistically significant.

## Results

### MgIG Attenuates MTX-Induced Intestinal and Liver Injuries

**Figure 1:**
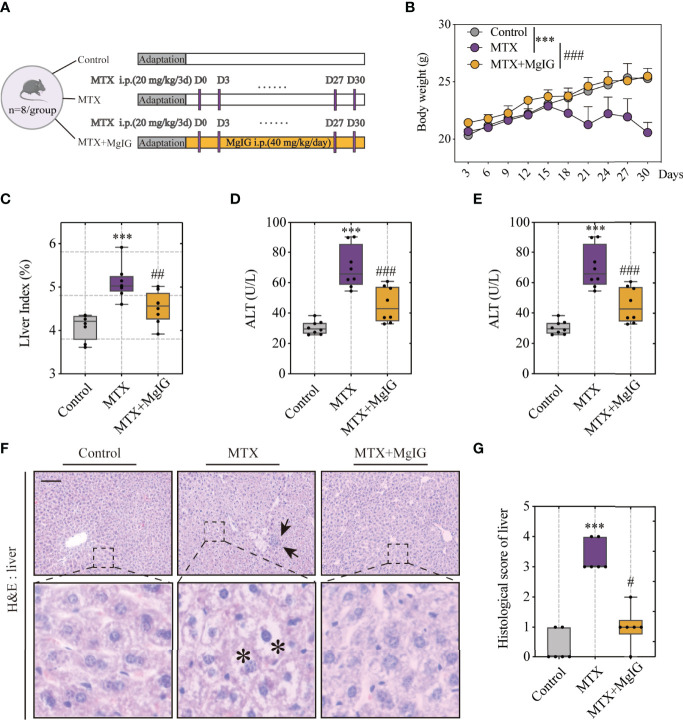
MgIG alleviated MTX-induced liver injury. **(A)** Experiment design. **(B)** Bodyweights during mouse model development (n = 8, Two-way ANOVA). **(C)** Liver index (liver weight/body weight) of each group (n = 8). **(D, E)** ALT and AST levels in serum(n = 8). **(F)** Representative H&E staining images of liver tissue from each group. Arrows and asterisks in the sections indicate histopathological differences between groups: inflammatory infiltration (arrow) and necrosis (asterisk). Scale bar, 100μm. **(G)** Histological score of liver tissue in each group (n = 6, Kruskal-Wallis test). ***p < 0.001, compared with Control group. ^#^p < 0.05, ^#^^#^p < 0.01, ^###^p < 0.001, compared with MTX group.

**Figure 2:**
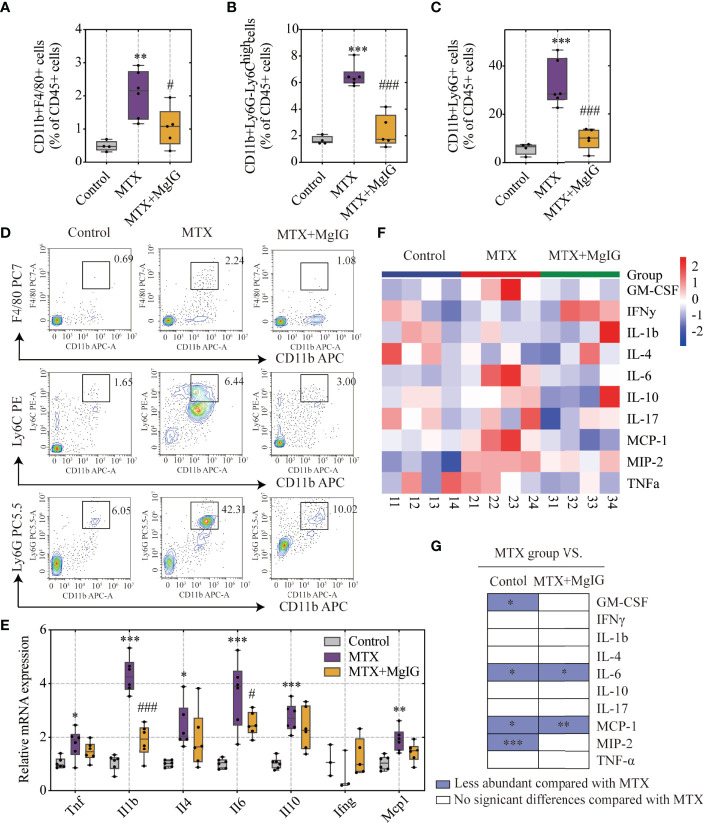
MgIG reduced MTX-induced inflammation levels. **(A–D)** FACS staining of immune cells (neutrophils, monocytes and macrophages) in the liver represented as percentages of CD45+ cells (n = 4-6, One-way ANOVA). **(E)** Relative mRNA expression of cytokines in liver tissues from each group (n = 3-6). **(F)** Heatmap of specific cytokine and chemokine levels in serum, including IFN-γ, IL-4, IL-6, GM-CSF, IL-1β, IL-17, IL-10, TNF-α, MIP-2, MCP-1 (n = 4). **(G)** Cytokines and chemokines from panel F and comparisons of MTX and other groups. Purple entries indicate cytokines/chemokines that were less abundant in various groups compared to the MTX group. *p < 0.05, **p < 0.01, ***p < 0.001, compared with Control group. ^#^p < 0.05, ^###^p < 0.001, compared with MTX group. ALT, alanine aminotransferase; AST, aspartate aminotransferase.

MgIG is widely used in the clinical treatment of liver disease. First, the attenuating ability of MgIG on MTX-induced liver and intestinal injuries was evaluated. C57BL/6 mice were administered 20 mg/kg/3d of MTX *via* an intraperitoneal injection, while a 5% glucose solution was administered to the control group. The concentration of MgIG was determined to be 40 mg/kg/day according to clinical administration (**Figure 1A**). MgIG significantly improved MTX-induced weight loss and liver index increase after 30 days of treatment (**Figures 1B, C**). Additionally, ALT and AST serum levels from biochemical tests were normalised on MgIG administration (**Figures 1D, E**), suggesting its hepatoprotective potential. The histological results also revealed that MTX-induced sinusoidal dilation, steatosis, fragmentary necrosis, and inflammatory cell infiltration in the liver were improved on MgIG intervention (**Figures 1F, G**). Inflammatory indicators in the liver after MgIG treatment were detected using flow cytometry and qRT-PCR, which found a significant decrease in inflammatory chemokines, cytokines (Tnf-α, Il-1β, Ifn-γ, Il-4 Il-6 and Il-10) (**Figure 2E**), and immune cells, including neutrophils, monocytes, and macrophages (**Figures 2A** **–D**). Furthermore, the levels of cytokines and chemokines in the serum were quantified using Luminex strategy, which showed that MTX triggered GM-CSF, IL-6, MCP-1, and MIP-2 increases (**Figures 2F, G**, **S1A**), which could be partly reversed on MgIG treatment.

**Figure 3:**
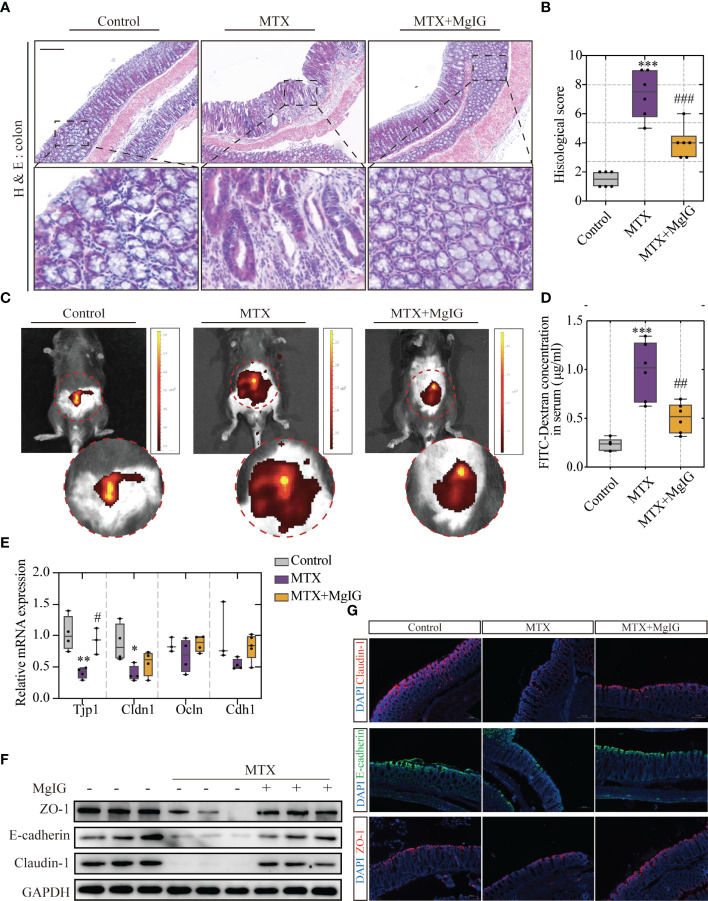
MgIG alleviated MTX-induced intestinal injury. **(A)** Representative H&E staining of colon tissue sections from each group, Scale bar, 200μm. **(B)** Histological score in each group (n = 6, Kruskal-Wallis test). **(C)** Representative *In vivo* imaging of FITC-Dextran. **(D)** Intestinal leakage measured by FITC-Dextran concentration in serum (n = 6, One-way ANOVA). **(E)** Relative mRNA expression of tight junctions in liver tissue from each group (n = 3-5). **(F)** Immunoblot analysis of ZO-1, E-cadherin and Claudin-1 in colon tissues (n = 3). **(G)** Immunofluorescence analysis on ZO-1, E-cadherin and Claudin-1 in colon sections from different groups. Representative images are shown. Scale bar, 100μm. *p < 0.05, **p < 0.01, ***p < 0.001, compared with Control group. ^#^p < 0.05, ^##^p < 0.01, ^###^p < 0.001, compared with MTX group.

According to our previous study, MgIG can also ameliorate chemotherapy-induced intestinal injury. Therefore, the effects of MgIG on intestinal injury inhibition, intestinal pathological status, and barrier structure were investigated. The histological examination of the colon tissue revealed epithelial damage, moderate goblet cell depletion, and evident mitosis at the intestinal crypts were significantly improved by MgIG. More inflammatory cell infiltration was observed in the colonic lamina propria (LP) and submucosa in MTX-treated mice compared to the control or MgIG-treated mice (**Figures 3A, B**). ‘Leak’ pathway, which is regulated by tight junctions, is often used to define intestinal permeability (26). Here in our research, fluorescein isothiocyanate (FITC)-dextran live images and quantification of fluorescence signal indicated that MgIG altered the intestinal leakage ability (**Figures 3C, D**). Moreover, tight junctions (ZO-1 and Claudin-1) and cell adhesion protein E-cadherin that maintain the intestinal barrier function of the colon were evaluated using immunofluorescence, western blot, and qRT-PCR, which showed that 30 days of intraperitoneal MTX injection caused a remarkable decrease in these tight junctions and adhesion protein expressions. By contrast, MgIG treatment resulted in an increase in the expression of these proteins (**Figures 3E–G**, **S1B–D**). These results suggest that MgIG alleviates both liver and gut injuries in MTX-treated mice models.

### MTX-Induced Hepatic Lesion Development Is Driven by Intestinal Barrier Dysfunction

**Figure 4:**
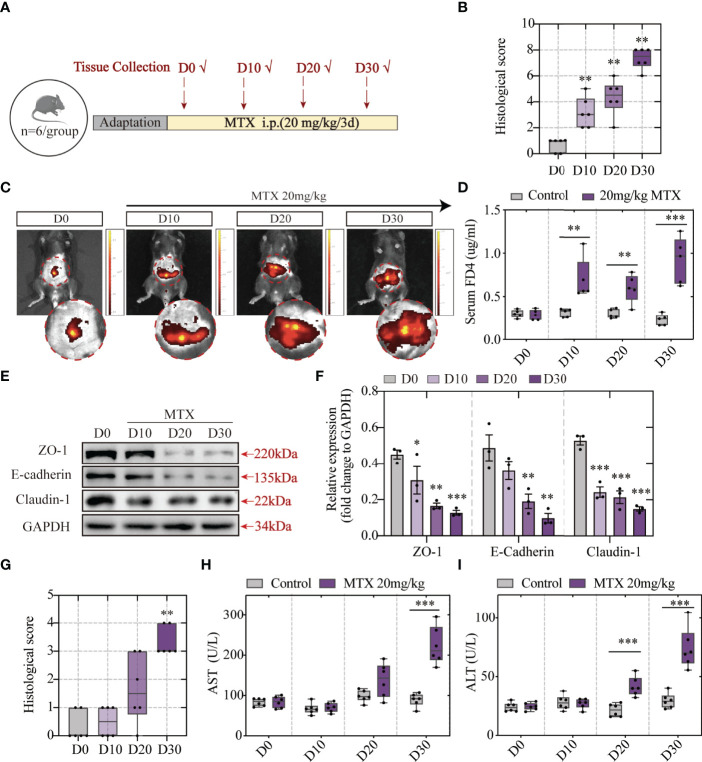
MTX-induced intestinal barrier disruption was detected in advance of hepatic lesion **(A)** Experiment design. **(B)** Histological score of colon tissue (n = 6, Kruskal-Wallis test). **(C)** Representative *in vivo* imaging of FITC-Dextran leakage. **(D)** Quantification of intestinal leakage measured by FITC-Dextran concentration in serum (n = 5, One-way ANOVA). **(E)** Immunoblot analysis of ZO-1, E-cadherin and Claudin-1 in colon sections from different groups (n = 3, One-way ANOVA). **(F)** Quantification of Figure 3E. **(G)** Histological score of liver tissue (n = 6, Kruskal-Wallis test). **(H, I)** ALT and AST levels in serum (n = 6, One-way ANOVA). *p < 0.05, **p < 0.01, ***p < 0.001, compared with D0 or Control.

To explore the association between intestinal barrier disruption and liver injury, the order of occurrence of hepatic lesion and intestinal barrier dysfunction was discussed. Tissue and blood samples were collected after MTX administration for 10/20/30 days and prepared for histological analyses and biochemical tests. The experiment design is elucidated in **Figure 4A**. The histological images revealed that MTX-treated mice presented first with intestinal injury rather than liver lesion. At the first time point, the tenth day, obvious inflammation was observed along with immune cells infiltration in colon (**Figures S2A**, **4B**). Till the last time point, serious epithelial damage and moderate goblet cell depletion were shown. Following this, the capacity of the FITC-dextran to extravasate was evaluated using *in vivo* fluorescent imaging system and verified using the quantification of dye leakage into the blood (**Figures 4C, D**). The expression of tight junctions and E-cadherin also tended to be lower as time goes on (**Figures 4E, F**). These results showed increased intestinal permeability after 10 days of MTX treatment and decreased tight junction expression, which resulted in increased leakage.

Haematoxylin and eosin (H&E) staining showed good cell integrity till 20 days. The presence of hepatic lesions was shown post 30 days of 20-mg/kg MTX administration (**Figures S2B**, **4G**). Consistent with H&E staining, biochemical tests of serum indicated that more than 20 days of MTX administration can lead to both ALT and AST concentration increase (**Figures 4H, I**). Therefore, MTX chemotherapy-related intestinal barrier disruption was speculated to drive hepatic lesion development because MTX-induced intestinal barrier disruption precedes hepatic lesion.

### Bacterial Translocation Is the Prerequisite of MTX-Induced Hepatic Lesion

**Figure 5:**
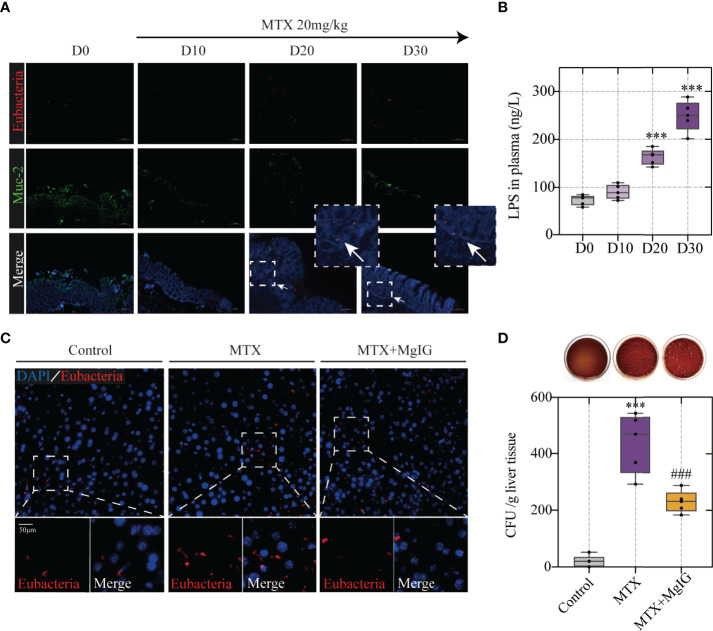
Bacterial translocation is the prerequisite of MTX-induced hepatic lesion **(A)** Representative images of Fluorescent *in situ* hybridization of the colon at different timepoints. Arrows indicate detected bacteria. **(B)** Lipopolysaccharide concentration in plasma (n = 5, One-way ANOVA). **(C)** Fluorescent *in situ* hybridization of the liver **(D)** Counting of colony-forming units in liver tissue of each group (n = 5, One-way ANOVA). ***p < 0.001, compared with D0 or Control group. ^###^p < 0.001, compared with MTX group.

Having observed increased intestinal permeability owing to increased tight junction expression and dextran leakage, the association between microbes or their metabolites, such as endotoxin translocation, and MTX in the colon was speculated. *Fluorescent in situ* hybridisation (FISH) analysis, using an EUB338I probe to mark the 16S rRNA region in eubacteria, was performed. Mucin (Muc-2) was used to indicate the intestinal mucus layer by immunofluorescence. The results showed that during MTX administration, bacteria were translocated from the intestinal mucus layer to the LP (**Figure 5A**), which correlated with increased lipopolysaccharide (LPS) translocation in the peripheral circulation (**Figure 5B**). As bacteria can enter the liver through the enterohepatic circulation, the bacteria in the liver were also evaluated using FISH analysis. MTX-treated mice livers harboured more bacteria than that found in controls, which were detected in the parenchyma (**Figure 5C**). The bacterial colonies in the liver were monitored by counting the colony-forming units (CFU) growing from the liver lysates. Bacteria could not be detected in the control group, whereas it existed at a dose of approximately 1 x 10^3 CFU in the MTX treatment group, directly proving bacterial migrating to the liver (**Figure 5D**).

Previous studies have shown that increase of LPS level, caused by bacteria localisation, might trigger an inflammatory process *via* interaction with its receptor toll-like receptor (TLR) 4 (27, 28).The infiltration of CD45+ leukocytes and expression of TLR4 in the liver were evaluated and showed an increase on MTX administration, suggesting TLR4-related inflammatory signalling pathway activation (**Figures S3A–E**). Macrophages represent a key cellular component of the liver that remove bacteria, toxins, and antagonizes infection, and are essential for maintaining tissue homeostasis and ensuring rapid responses to hepatic injury (29). Moreover, microbiota-derived TLR ligands have been reported to modulate the development and function of extra-intestinal macrophages (M1/M2) (4, 30). Therefore, multiple immunofluorescence staining was used to assess M1/M2 polarisation, which showed that MTX administration promoted proinflammatory M1 (CD68+iNOS+) macrophage phenotype but attenuated typical M2 (CD68+CD163+) macrophage traits in the liver (**Figures S3F–H**). These results confirmed that MTX-induced liver inflammation was caused by enterobacteria translocation.

However, MgIG intervention remarkably down-regulated TLR4-related pathway and increased M2 macrophage phenotype, owing to reduced bacterial colonies and decreased LPS levels.

### MgIG Protects the Gut Barrier by Altering the Gut Microbial Composition and Increasing *Lactobacillus* Levels

**Figure 6:**
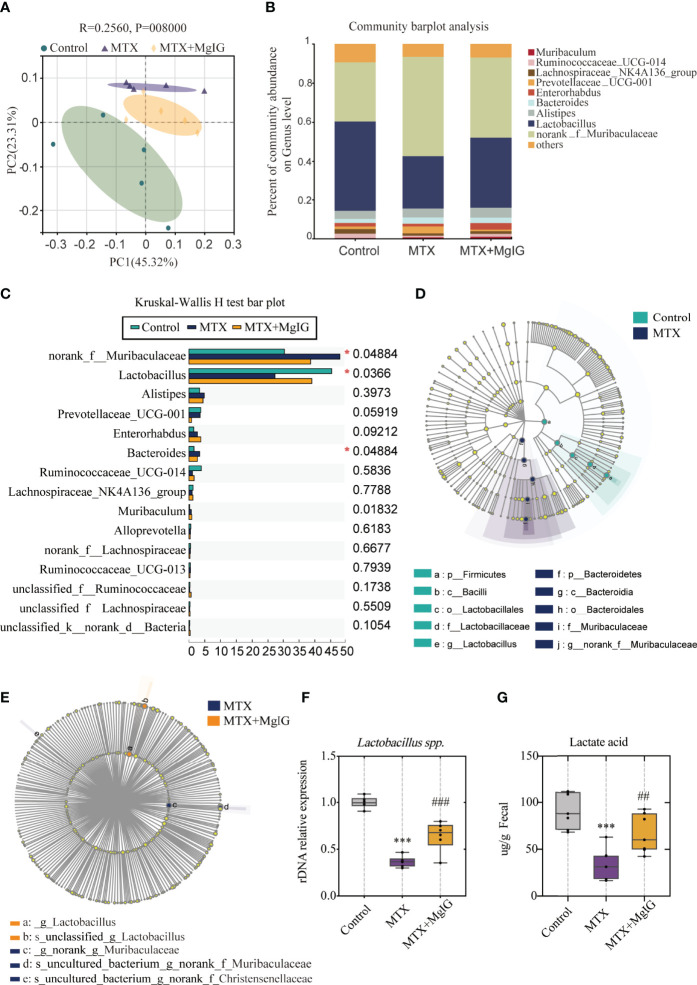
16s rDNA sequencing revealed altered microbiota composition after MTX or MgIG treatment **(A)** PCoA analysis with the ANOSIM significant test for each group (n = 5). **(B)** Relative abundance of microbial taxa was determined at the genus level. Top 10 abundances are shown. **(C)** Significance test of multiple groups using Kruskal-Wallis H test. **(D, E)** Multilevel species-level bar chart from LEfSe analysis, showing biomarker taxa at the genus level (LDA score) of >4 and a significance of P < 0.05 determined by the Wilcoxon signed-rank test. **(F)** 16s rDNA relative expression measured by quantitative PCR. **(G)** Fecal lactic acid concentration of each group (n = 6). ***p < 0.001, compared with Control group. ^##^p < 0.01, ^###^p < 0.001, compared with MTX group.

Gut microbiota alterations have been reported to contribute to intestinal epithelial homeostasis and drive intestinal injury development (31, 32). MgIG is considered to have low bioavailability owing to its low absorption rate. Thus, we deliberated the indirect protective effect of MgIG. Recently, the advances in multi-omics sequencing has allowed researchers to investigate the complexity of the intestinal microbiota in various human diseases (33). In this study, we used 16s ribosomal RNA gene sequencing to explain the underlying mechanism of the intestinal barrier repairment on MgIG treatment, which could be achieved by altering gut microbiota composition. Principal coordinates analysis (PCoA) revealed distinct microbiota composition clusters for each group (**Figure 6A**). Community barplot analysis described the detailed microbiota composition and proportion of each species, and top 10 abundances were shown (**Figure 6B**).

First, Kruskal-Wallis H test was conducted. This analysis allows for significant differences in species across multiple groups of samples. From the results, abundance of *Muribaculaceae, Lactobacillus*, and *Bacteroides* present marked differences among three groups (**Figure 6C**). Linear discriminant analysis Effect Size (LEfSe) analysis was performed, with an LDA score > 4 used to select the genera, to identify the altered bacteria after MTX or MgIG treatment. Notably, MTX down-regulated the bacterial genus *Lactobacillus* but up-regulated *Muribaculaceae* compared with the control group (**Figures 6D**, **S4A**). However, MgIG treatment showed the opposite changes in microbial composition (**Figure 6E**, **S4B**). *Lactobacillus* alteration was further confirmed in rDNA level using relative quantitative PCR (**Figure 6F**). These analyses revealed that MgIG treatment significantly increased *Lactobacillus*. Additionally, the increase of lactic acid level in faeces was observed as well on MgIG treatment (**Figure 6G**).

*Muribaculaceae*, also called S24-7, has only been recently cultured, thereby posing a difficulty in studying its correlation with the host. Recent studies have revealed that *Muribaculaceae* may be associated with the functionality of the intestinal barrier (34, 35). However, its exact mechanism and influence remain unclear. *Muribaculaceae* has been associated with decreased mucin expression and LPS production in some cases, whereas it has been associated with probiotic properties in other cases. This study revealed that *Muribaculaceae* abundance was negatively correlated with mucus layer thickness and positively correlated with increased intestinal permeability using periodic acid-schiff (PAS) staining and immunofluorescence staining of muc-2 (**Figures S5A, B**).

The up-regulated *Lactobacillus*, a well-known probiotic, was recently reported to improve metabolic impairments and alcoholic liver disease *via* intestinal barrier function improvement (36, 37). However, the underlying mechanism remains unclear. Furthermore, 16s rRNA sequencing showed that *Lactobacillus* depletion induced by MTX was reversed on MgIG treatment, suggesting its potential beneficial role in DILI treatment. Additionally, the change in *Bacteroides* abundance was consistent with that of *Lactobacillus*. Accumulative evidence has shown that *Lactobacillus* and *Bacteroides* can promote short-chain-fatty-acids (SCFA) generation. Therefore, GasChromatography-MassSpectrometer (GC-MS) analyses of the SCFA levels in faeces showed that MgIG reversed the MTX-induced SCFAs’ decline to some extent (**Figure S5C**).

### *Lactobacillus* Prevents Intestinal Barrier Impairment and Liver Damage in MTX-Induced Mice

Based on the sequencing result, *Lactobacillus* was hypothesised to protect MTX-induced liver injury by improving the intestinal barrier function, which may explain the hepatoprotective function of MgIG.

**Figure 7:**
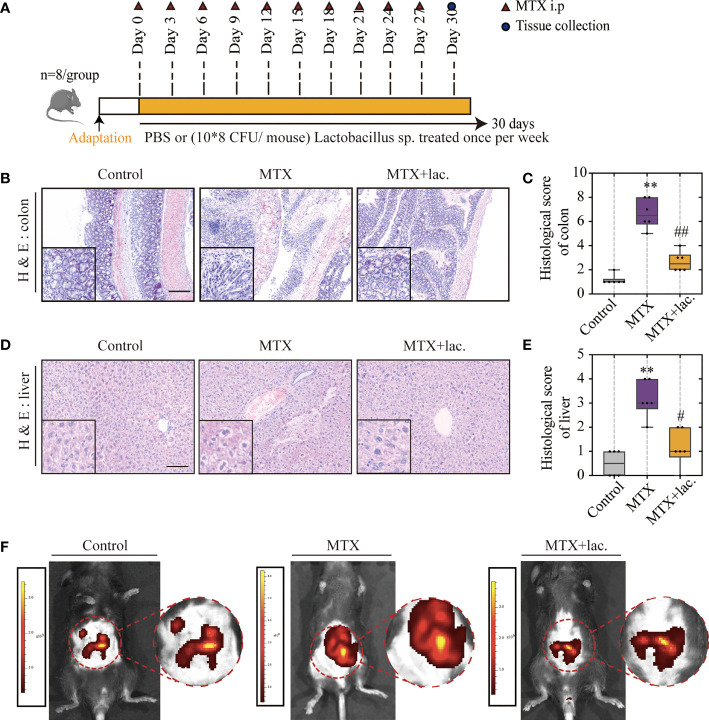
*Lactobacillus.* sp. can protect the liver and intestine against MTX-induced toxicity. **(A)** Experiment design. **(B, C)** Representative H&E staining of liver tissues and histological score, Scale bar, 100μm. **(D, E)** Representative H&E staining of colon tissue sections and histological score in each group, Scale bar, 200μm (n=6, Kruskal-Wallis test). **(F)** *In vivo* imaging of FITC-Dextran leakage. **p < 0.01, compared with Control group. ^#^p < 0.05, ^##^p < 0.01, compared with MTX group.

**Figure 8:**
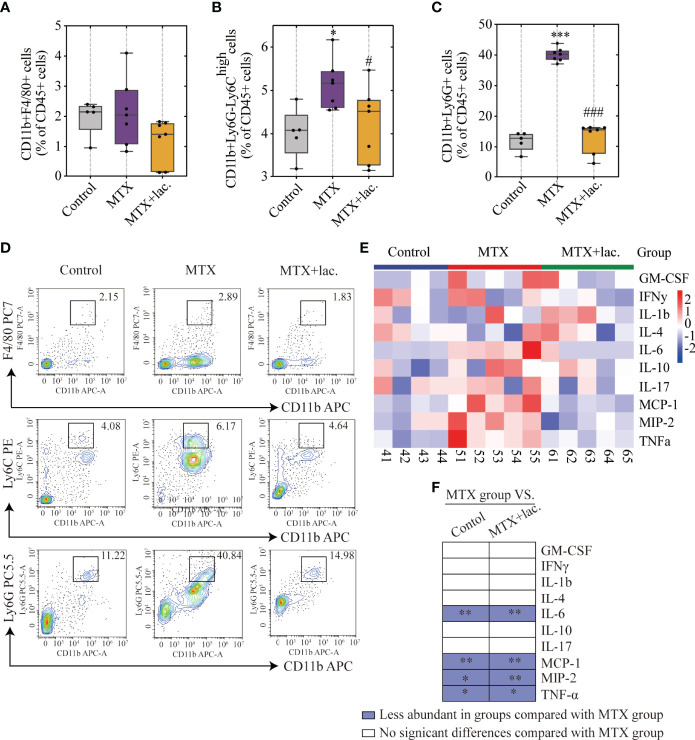
*Lactobacillus.* sp. reduced MTX-induced inflammation levels in liver and serum. **(A–D)** FACS staining of immune cells in the liver represented as percentages of CD45+ cells. **(E)** Heatmap of specific cytokine and chemokine levels in serum, including IFN-γ, IL-4, IL-6, GM-CSF, IL-1β, IL-17, IL-10, TNF-α, MIP-2, MCP-1 (n=4-5). **(F)** Cytokines and chemokines from panel L and comparisons of MTX and other groups. Blue entries indicate cytokines/chemokines that were less abundant in various groups compared to the MTX group. *p < 0.05, **p < 0.01, ***p < 0.001, compared with Control group. ^#^p < 0.05, ^###^p < 0.001, compared with MTX group.

To confirm this hypothesis, *Lactobacillus* sp. were cultured in Man, Rogosa, and Sharpe (MRS) broth *ex vivo*, and mice were orally administrated with *Lactobacillus* sp. (10^8^ CFU/mouse) once a week for a total of 4 weeks (**Figure 7A**). Similar to MgIG, *Lactobacillus* sp. were efficient against MTX-induced liver and intestinal pathological injuries (**Figures 7B** **–E**), with significant improvement in intestinal leakage, which could be attributed to the intestinal barrier repair (**Figure 7F**). *Lactobacillus* sp. prevented the infiltration of monocytes and neutrophils, but the proportion of F4/80+ macrophages were not changed (**Figures 8A** **–D**). *Lactobacillus* sp. also suppressed the LPS-related TLR4 inflammatory pathway (**Figures S6B, C**), which suppressed liver inflammation. Additionally, inflammation indicators (IL-6, MCP-1, MIP-2 and TNF-α) in the serum were down-regulated from luminex (**Figures 8E, F**, **S6A**).

### MgIG Restores the Intestinal Barrier Function and Ameliorates Hepatic Injury in a Gut-Microbiota Dependent Manner

**Figure 9:**
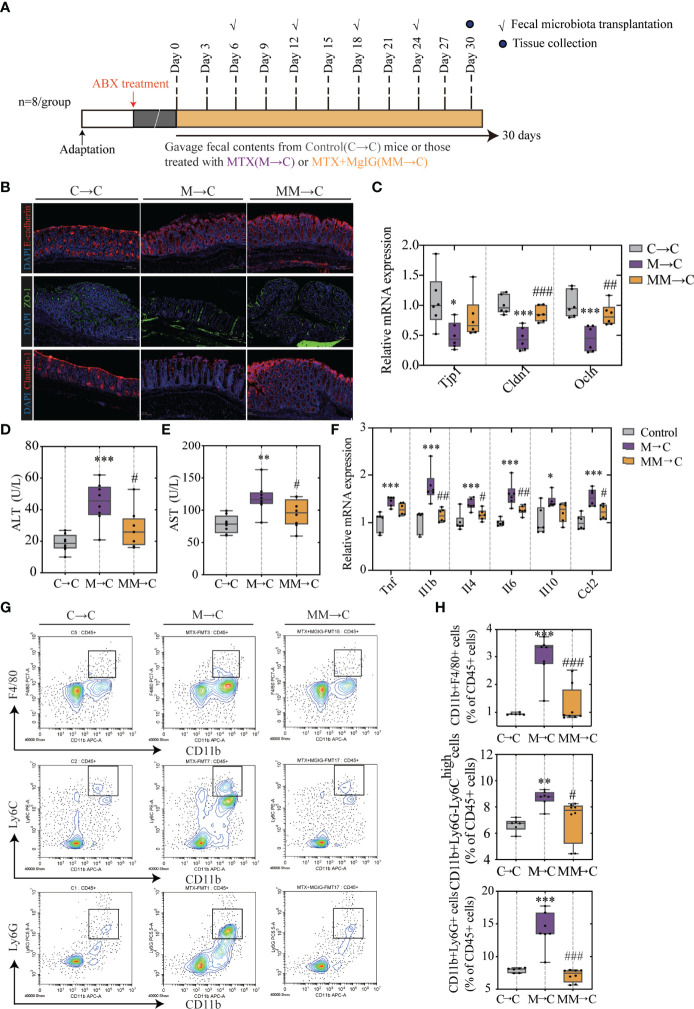
FMT helped restoring intestinal barrier function and ameliorating hepatic inflammation. **(A)** Experiment design. **(B)** Immunofluorescence analysis on ZO-1, E-cadherin and Claudin-1 in colon sections from different groups. Representative images are shown. Scale bar, 100μm. **(C)** Relative mRNA expression of tight junctions and cell adhesion protein in colon. **(D, E)** ALT and AST levels in serum (ALT, n = 6. AST, n = 4, One-way ANOVA). **(F)** Relative mRNA expression of cytokines in liver (n = 6, One-way ANOVA). **(G, H)** Quantification of immune cells in the liver represented as percentages of CD45+ cells (n = 6-8). *p < 0.05, **p < 0.01, ***p < 0.001, compared with Control group. ^#^p < 0.05, ^##^p < 0.01, ^###^p < 0.001, compared with M→C group. FMT, fecal microbiota transplantation.

Faecal microbiota transplantation (FMT) was used to verify the association between microbiota and MTX-induced intestinal and liver injuries. Faecal microbiota from MgIG treated or untreated MTX models were transplanted into recipients who were pre-processed with an antibiotic cocktail (**Figure 9A**).

As displayed in **Figures 9B, C**, compared with the MTX-donor FMT, MgIG-FMT showed alleviation in intestinal leakage. The expression of ZO-1, claudin-1, and E-cadherin was significantly increased in the MgIG-FMT group, suggesting the restoration of the mucus layer thickness (**Figures 9B, C**). Furthermore, MTX-FMT resulted in abnormal ALT or AST level (**Figures 9D, E**), and induced palpable liver inflammation, including leakage of immune cells and increase of inflammatory cytokines (**Figures 9F** **–H**). However, these inflammatory responses were lower in the MgIG-FMT mice. Therefore, the microbiota can help MgIG to prevent intestinal and liver injuries.

## Discussion

The human intestinal tract contains diverse microbes, which have established a symbiotic relationship with the host (38). Recent studies have defined their correlation with various liver disease, with links to disease pathogenesis and use as therapeutic targets (39–41). Alteration in the gut microbiota composition have been reported to cause intestinal barrier disruption, which can induce bacterial and bacterial product translocation to the systemic circulation and bacterial colony formation in the liver, resulting in liver injury (42, 43). Intestinal barrier disruption has been postulated to have various critical roles in the development of different liver disorders; however, its role in DILI remains unclear. In this study, we used the commonly administrated chemotherapy drug MTX to explore the relationship between drug-induced intestinal and liver injuries. The disruption of the structure and function of the intestinal mechanical barrier is an early event in MTX-DILI pathogenesis, owing to microbial disorder, including decreased abundance of probiotics and increased abundance of harmful bacteria. Gut bacteria are successfully translocated from the intestinal lumen into the LP, triggering an inflammatory response both in the gut and liver. Therefore, intestinal health could be used as a target for a novel preventative treatment method for MTX-related DILI.

MgIG is synthesised from 18-β glycyrrhizic acid, which is the main component of *Glycyrrhiza*, commonly used in traditional Chinese medicine. Although its protective effect on MTX-induced liver toxicity has been well documented, the underlying mechanism remains unclear (44). Previous studies have found that the alleviation of the intestinal barrier injury caused by MTX can be attributed to the treatment of MgIG (22). MgIG has been hypothesised to defend against MTX-related DILI through the gut–liver axis. Accordingly, this study demonstrated that MgIG treatment restored bacterial composition alteration, thereby repairing the intestinal barrier and inhibiting bacterial translocation. The use of 16s rRNA sequencing in this study identified possibly altered bacterial species and found that *Lactobacillus* abundance was negatively correlated with increased intestinal permeability, suggesting the intestinal protective effect of *Lactobacillus. Lactobacillus* was recently reported to prevent alcoholic liver disease through intestinal barrier function improvement, owing to its probiotic characteristics. Additionally, it is involved in the protection of the liver in DILI, which was validated using *in vitro* cultures and gavage on MTX-induced model. *Muribaculaceae*, a type of LPS-producing bacterium, was found to positively correlate with intestinal permeability and liver inflammation; however, further validation and investigation are required owing to its unknown sequence. The use of FMT to verify the regulating ability of gut microbiota after MgIG treatment showed that FMT ameliorated liver inflammation and decreased intestinal permeability. Therefore, this study provides evidence for the anti-DILI effects of MgIG in MTX-treated mouse models. Additionally, maintaining gut health *via* MgIG treatment or specific prebiotic supplementation, such as *Lactobacillus*, can be considered as a novel strategy for DILI treatment.

## Conclusion

This study revealed that MgIG remarkably attenuated MTX-induced intestinal and liver injuries *via* regulation of the intestinal barrier function. We found that chronic administration of MTX caused bacterial translocation and lead to liver inflammation, whereas MgIG can restore the intestinal barrier *via* altering the gut microbial composition. It retrieved up-regulated *Lactobacillus* but down-regulated *Muribaculaceae* after MTX administration, and on the contrary, MgIG increased *Lactobacillus* abundance and decreased *Muribaculaceae* abundance. Furthermore, similar to MgIG, *Lactobacillus* supplement can protect liver and intestines against MTX. Collectively, in this study, we elucidated the mechanism and therapeutic effect of MgIG on the liver from the perspective of the gut–liver axis and identified a new treatment approach for MTX-induced intestinal and liver injuries.

## Data Availability Statement

The original contributions presented in the study are publicly available. This data can be found here: https://www.ncbi.nlm.nih.gov/search/all/?term=PRJNA791793.

## Ethics Statement

The animal study was reviewed and approved by Animal Care and Use Committee of Nanjing University of Chinese Medicine.

## Author Contributions

YL and ZW designed the study and supervised all parts of the project. YX and HS conducted the whole study. CQ and HH performed the molecular biology experiments. KL and RT conducted animal experiments. YX and HS did the bioinformatics analyses in close collaboration with ZW drafted the first versions. RG and YZ contributed to text revision and discussion. All authors read and approved the final manuscript.

## Funding

This work was financially supported by National Natural Science Foundation of China (82004124, 81961128020, 81973734); Natural Science Foundation of Jiangsu Province (BK20200154); China Postdoctoral Science Foundation (2020M671551). This project was supported in part by the Open Project of Chinese Materia Medica First-Class Discipline of Nanjing University of Chinese Medicine (2020YLXK20).

## Conflict of Interest

The authors declare that the research was conducted in the absence of any commercial or financial relationships that could be construed as a potential conflict of interest.

## Publisher’s Note

All claims expressed in this article are solely those of the authors and do not necessarily represent those of their affiliated organizations, or those of the publisher, the editors and the reviewers. Any product that may be evaluated in this article, or claim that may be made by its manufacturer, is not guaranteed or endorsed by the publisher.

## Abbreviations

MTX, methotrexate; ALL, acute lymphoblastic leukaemia; ALT, alanine aminotransferase; AST aspartate aminotransferase; RA, rheumatoid arthritis; PsA, psoriatic arthritis; MgIG, magnesium isoglycyrrhizinate; DILI,drug-induced liver injury; NAFLD, non-alcoholic fatty liver disease; H&E, haematoxylin and eosin; FISH, fluorescent *in situ* hybridisation; LPS, lipopolysaccharide; CFU, colony-forming units; TLR, toll-like receptor; PCoA, Principal coordinates analysis; LEfSe, Linear discriminant analysis Effect Size; PAS, periodic acid-schiff; SCFA, short-chain-fatty-acids; GC-MS, Gas Chromatography-Mass Spectrometer; MRS, Man, Rogosa, and Sharpe; FMT, Faecal microbiota transplantation; SPF, specific-pathogen-free facility; OTUs, operational taxonomic units; QIIME, Quantitative Insights into Microbial Ecology; PBS, phosphate-buffered saline; PVDF, polyvinylidene fluoride; TBST, tris-buffered saline with 0.1% Tween 20; HRP, Horseradish Peroxidase; EDTA, ethylenediaminetetraacetic acid; SD, standard deviation.
