# Erratum: Modulation of Gut Microbiota by Magnesium Isoglycyrrhizinate Mediates Enhancement of Intestinal Barrier Function and Amelioration of Methotrexate-Induced Liver Injury

**DOI:** 10.3389/fimmu.2022.976502

**Published:** 2022-07-04

**Authors:** Frontiers Production Office

**Affiliations:** Frontiers Media SA, Lausanne, Switzerland

**Keywords:** methotrexate, magnesium isoglycyrrhizinate, gut-liver axis, bacterial translocation, gut microbiota

Due to a production error, the Acknowledgements section was not included in the article. The acknowledgements section should read:

“ The authors thank Nanjing Jiangbei New Area Biopharmaceutical Public Service Platform for the quantification of Short-Chain Fatty Acids. Many thanks to Pro. Lu, our academic and life mentor, for strong spiritual support.”

The original version of this article has been updated.

