# Supplementary material for: Modulation of Gut Microbiota by Magnesium Isoglycyrrhizinate Mediates Enhancement of Intestinal Barrier Function and Amelioration of Methotrexate-Induced Liver Injury
Source: Front Immunol. 2022 May 12;13:874878. doi: 10.3389/fimmu.2022.874878 (PMC9134347; doi:10.3389/fimmu.2022.874878)
Supplement: Supplementary file 1 [file DataSheet_1.pdf]

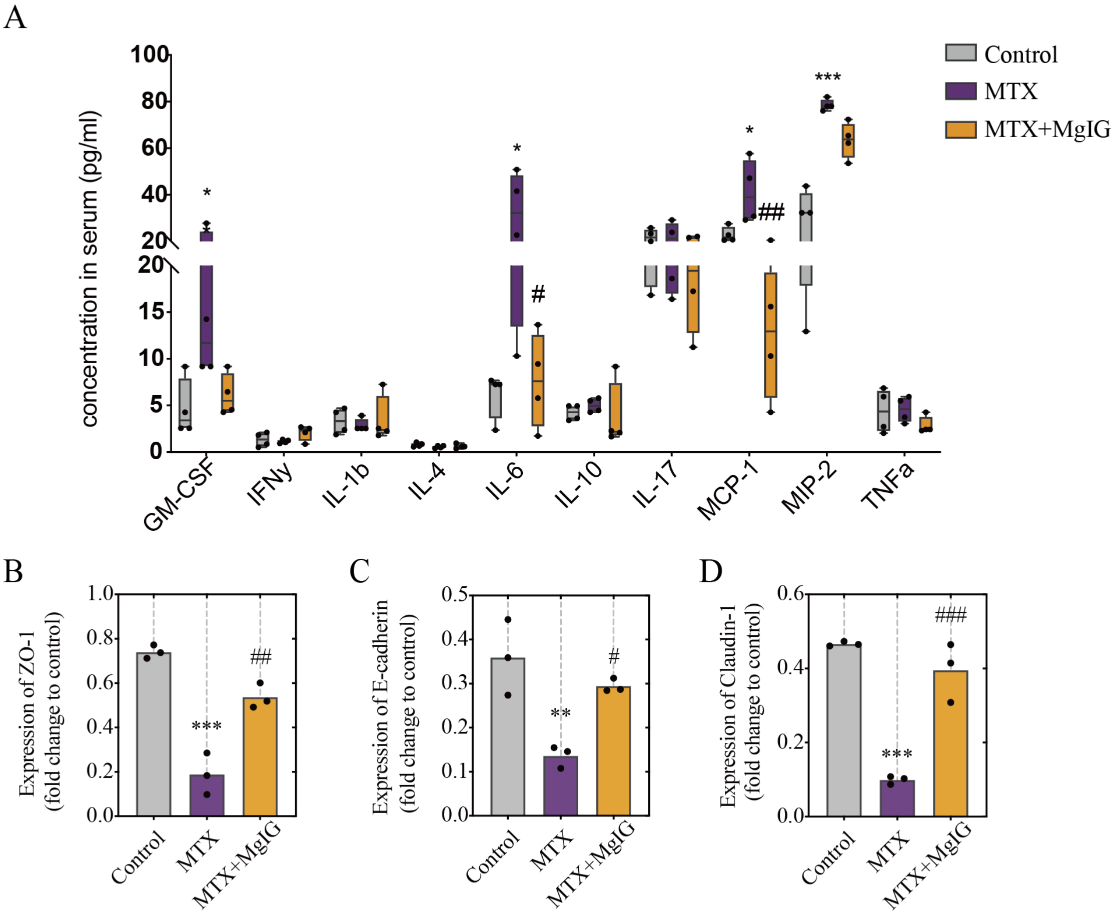

Supplementary Figure 1. MgIG suppressed MTX-induced inflammation and intestinal barrier disruption. (A) Histogram of luminex analysis (n=4). (B-D) Quantification analysis of tight junction and E-cadherin (n=3).

*p<0.05, **p<0.01, ***p<0.001, compared with Control group. #p<0.05, ##p<0.01, ###p<0.001, compared with MTX group.

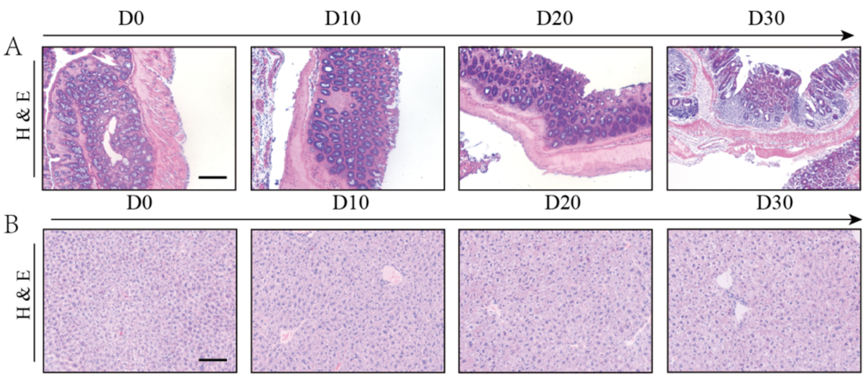

Supplementary Figure 2. MTX-induced intestinal barrier disruption occurred before hepatic lesion

(A-B) Representative H&E staining of liver and colon tissues from different timepoints.

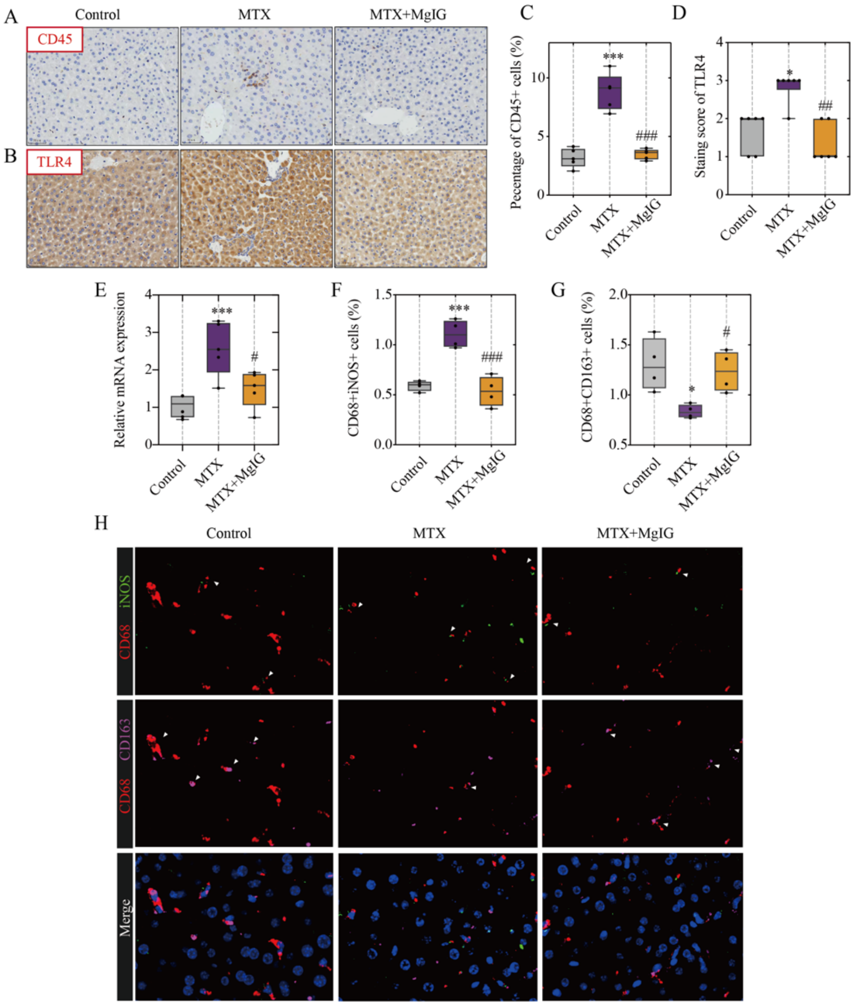

Supplementary Figure 3. MgIG inhibited the activation of LPS-related inflammatory signaling pathway caused in the liver (A) Immunohistochemical staining of CD45+ leukocytes. (B) Immunohistochemical staining of TLR4 expression in the liver. (C) Quantification of the percentage of CD45+ cells. (D) Quantification of staining score of TLR4 according to the color depth. (E)Relative mRNA expression of Tlr4 in liver tissue (n=6). (F-G) Proportion of CD68+iNOS+ or CD68+CD163+ macrophages. (H) Representative multiple immunofluorescence staining from each group. Arrows indicate positive cells.

*p <0.05, ***p<0.001, compared with Control group. #p<0.05, ##p<0.01, ###p<0.001, compared with MTX group.

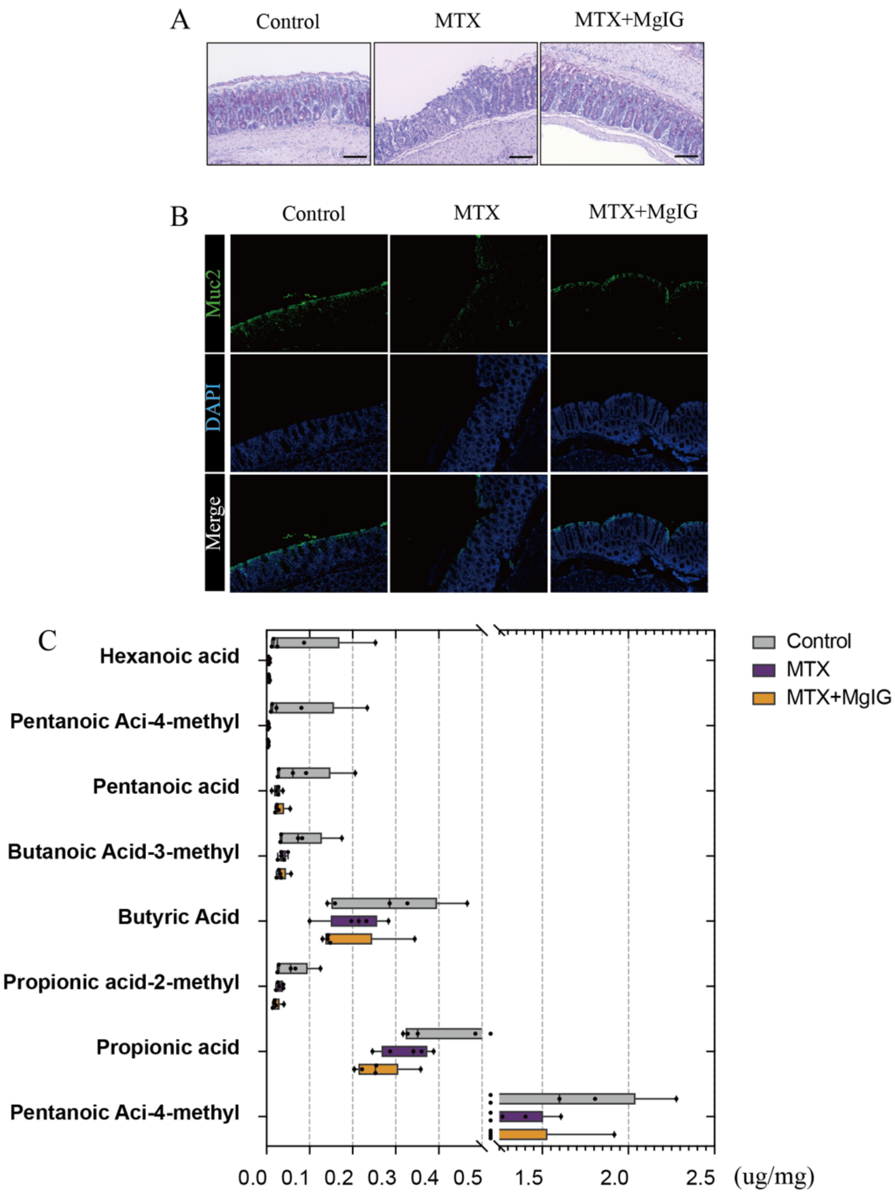

Supplementary Figure 4. MgIG restored the intestinal mucus layer (A) Periodic Acid-Schiff staining images from each group. (B) Immunofluorescence of Mucin-2 of colon sections. (C) SCFAs level in feces measured by GC-MS.

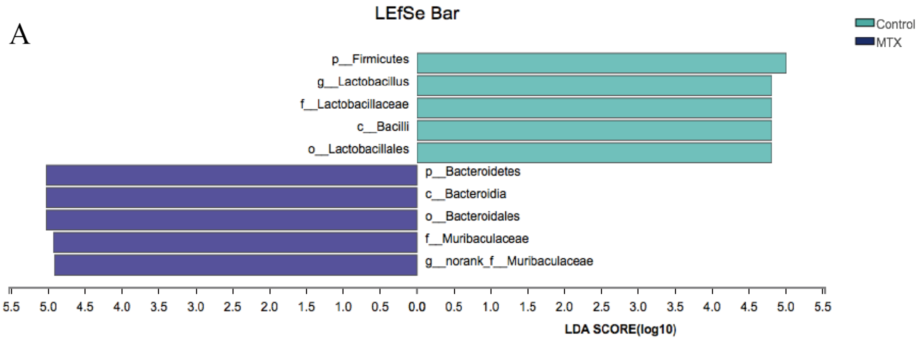

Supplementary Figure 5. MgIG altered the gut microbial composition (A-B) Linear discriminant analysis (LDA) scores derived from LEfSe analysis, showing biomarker taxa at the genus level (LDA score) of >4 and a significance of P <0.05 determined by the Wilcoxon signed-rank test. A presented the difference between control and MTX group. B presented the difference between MTX and MTX+MgIG group.

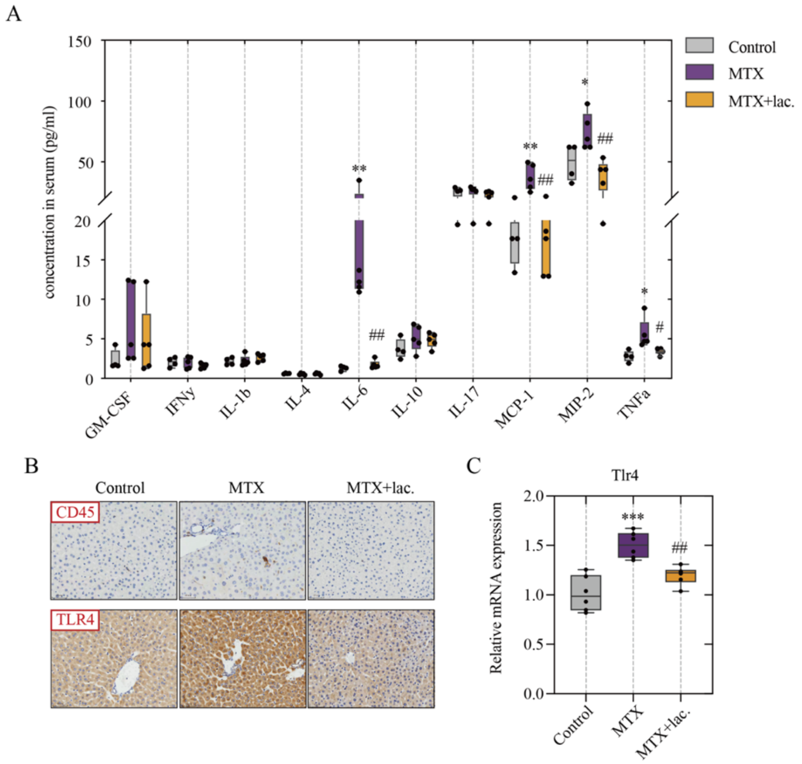

Supplementary Figure 6. *Lactobacillus. sp.* inhibited the activation of LPS-related inflammatory signaling pathway caused in the liver (A) FACS staining results of immune cells (neutrophils, monocytes and macrophages) infiltration indicated by the percentage of CD45+ cells. (B) Immunohistochemical staining of CD45+ leukocytes. (C) Immunohistochemical staining of TLR4 in the liver. (D) Relative mRNA expression level of TLR4 (n=6). (E) Histogram of luminex analysis. *p<0.05, **p<0.01, ***p<0.001, compared with Control group. #p<0.05, ##p<0.01, compared with MTX group.

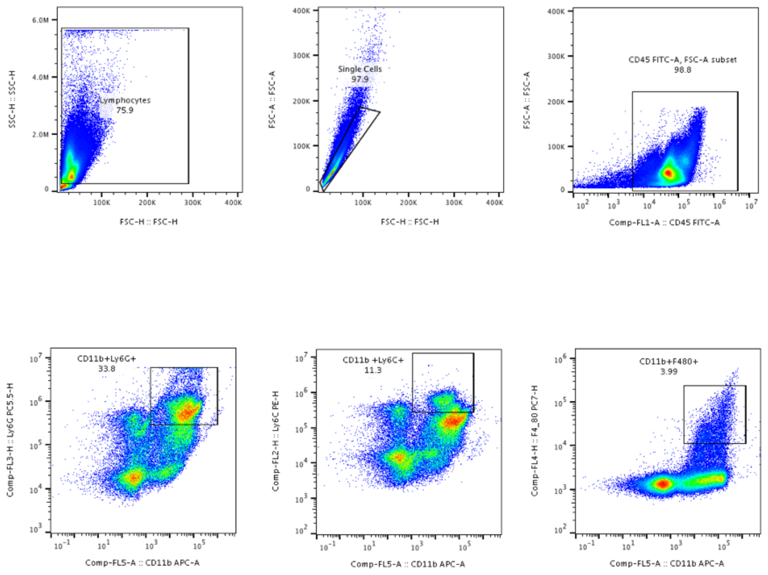

Supplementary Figure 7. Gating methods of cytometry figures (Fig 2D, 8D, 9G)

Supplementary Table 1.

Table 1. Ishak grading score system of histopathological assessment

Histology Characteristics		Grading Score
A	piecemeal necrosis	0-4
B	confluent necrosis	0-6
C	focal necrosis	0-4
D	portal inflammation	0-4

*Total score=A+B+C+D. The maximum grading score is 18.
